# Vitamin D Actions: The Lung Is a Major Target for Vitamin D, FGF23, and Klotho

**DOI:** 10.1002/jbm4.10569

**Published:** 2021-11-18

**Authors:** Ghislaine Gayan‐Ramirez, Wim Janssens

**Affiliations:** ^1^ Laboratory of Respiratory Diseases and Thoracic Surgery (BREATHE), Department CHROMETA KU Leuven Leuven Belgium; ^2^ Clinical Department of Respiratory Diseases UZ Leuven Leuven Belgium

**Keywords:** CELL/TISSUE SIGNALING–ENDOCRINE PATHWAYS, PTH/VIT D/FGF23, OTHER, THERAPEUTICS

## Abstract

Vitamin D is well known for its role as a calcium regulator and in maintenance of phosphate homeostasis in musculoskeletal health, and fibroblast growth factor 23 (FGF23) and its coreceptor α‐klotho are known for their roles as regulators of serum phosphate levels. However, apart from these classical actions, recent data point out a relevant role of vitamin D and FGF23/klotho in lung health. The expression of the vitamin D receptor by different cell types in the lung and the fact that those cells respond to vitamin D or can locally produce vitamin D indicate that the lung represents a target for vitamin D actions. Similarly, the presence of the four FGF receptor isoforms in the lung and the ability of FGF23 to stimulate pulmonary cells support the concept that the lung is a target for FGF23 actions, whereas the contribution of klotho is still undetermined. This review will give an overview on how vitamin D or FGF23/klotho may act on the lung and interfere positively or negatively with lung health. © 2021 The Authors. *JBMR Plus* published by Wiley Periodicals LLC on behalf of American Society for Bone and Mineral Research.

## 1 Introduction

The vitamin D‐fibroblast growth factor 23 (FGF23)‐klotho axis is well known for its role in regulating calcium and phosphate homeostasis. Interestingly, its role in targeting the lung has been the focus of several studies during the past decades. Particular attention has been paid to the implication of vitamin D in lung health, and more recently, FGF23 has emerged as a potential player in lung inflammation, while the role of klotho, its coreceptor, has not yet been determined in lung.

Although vitamin D has been, for a long time, essentially viewed as a regulator of calcium and phosphate homeostasis and musculoskeletal health, increased awareness has emerged on the plurality of vitamin actions in regulating many other organ systems, including the lung. The discovery that different cell types in the lung express the vitamin D receptor (VDR)^(^ ^1^, ^2^, ^3^, ^4^, ^5^, ^6^, ^7^ ^)^ and respond to 1,25 dihydroxyvitamin D has supported the concept that the lung represents a target for vitamin D actions. Importantly, vitamin D exerts its actions on the lung from fetal development throughout life, with any vitamin D dysfunction during fetal development having long‐term consequences on lung health after birth. Moreover, several cell types in the lung express the activating 1α‐hydroxylase (CYP27B1)^(^ ^2^, ^3^, ^8^, ^9^ ^)^ and are thus able to locally produce 1,25 dihydroxyvitamin D that will exert its actions in an autocrine or paracrine fashion. The understanding of the potential impact of FGF23 on lung is in its infancy, but the few available evidences underline a possible role in lung inflammation. In its classical actions, FGF23 can directly target different cell types in a variety of organs and its actions are mediated by specific FGF receptor isoforms requiring or not its coreceptor klotho.^(^ ^10^ ^)^ The lung expresses the four FGFR isoforms,^(^ ^11^, ^12^ ^)^ but whether klotho is expressed in the lung is still controversial.^(^ ^12^, ^13^, ^14^, ^15^, ^16^, ^17^, ^18^, ^19^, ^20^, ^21^, ^22^, ^23^, ^24^ ^)^

The goal of this review is to document the current evidences for the role of the vitamin D‐FGF23/klotho axis on the lung. The review will first briefly summarize the metabolism of vitamin D and FGF23 to facilitate the understanding of the review. It will then focus on the thematic of the review by giving an overview on how the vitamin D and FGF23 (klotho) actions can target the lung. In particular, it will highlight which cells in the lung are potential targets for vitamin D or FGF23 and how relevant are these actions starting in fetal lung development and continuing during the whole life.

## 2 Interfered Regulation/Metabolism of Vitamin D and FGF23/Klotho

**Fig 1:**
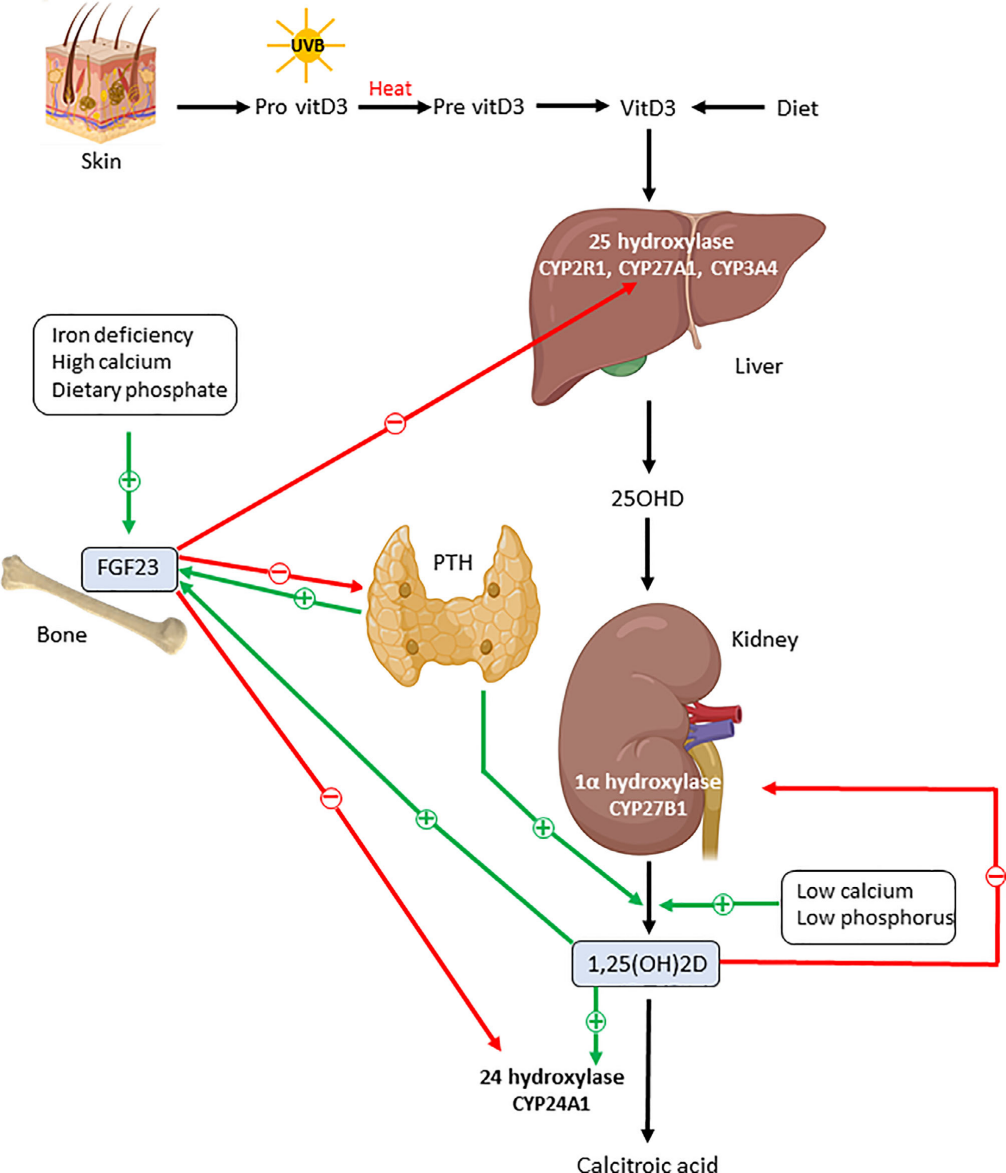
Schematic overview of the metabolism and regulation of vitamin D and FGF23. Icons have been designed by Biorender. See text for details.

Vitamin D is a fat‐soluble secosteroid, primarily produced in skin after exposure to sunlight and converted to active vitamin D after two hydroxylation steps: one occurring in the liver to produce 25‐hydroxyvitamin D (25OHD) by 25‐hydroxylases (CYP2R1, CYP27A1, CYP3A4), and the second taking place in the kidneys where 25OHD is converted by CYP27B1 into the biologically active 1,25‐dihydroxyvitamin D (1,25(OH)2D.^(^ ^25^, ^26^ ^)^ FGF‐23, which belongs to the FGF19 subfamily that functions as circulating hormones,^(^ ^27^, ^28^ ^)^ is principally produced in osteocytes and osteoblasts^(^ ^29^ ^)^ (Fig. 1). {FIG1}

The renal synthesis of 1,25(OH)2D is tightly regulated by serum calcium, phosphorus, FGF23, and regulating hormones, such as parathyroid hormone (PTH), calcitonin, and phosphatonins, while a negative feedback is provided by 1,25(OH)2D itself through downregulation of CYP27B1.^(^ ^25^ ^)^ FGF23 production is regulated by factors related to mineral metabolism with dietary phosphate consumption and 1,25(OH)2D representing the main stimuli of FGF23 production,^(^ ^30^, ^31^ ^)^ which is also directly stimulated by hypercalcemia^(^ ^32^ ^)^ and PTH and also indirectly by PTH through increased 1,25(OH)2D.^(^ ^29^ ^)^ Several factors related to pathological situations are also able to stimulate FGF23 production. These include iron deficiency only in situations of impaired FGF23 cleavage,^(^ ^33^ ^)^ systemic inflammation via a direct effect on osteocytes and indirectly mainly by regulating iron metabolism or by altering systemic calcium, phosphate, and vitamin D metabolism,^(^ ^34^ ^)^ and finally hypoxia‐inducible factor (HIF)‐1α, which is induced in osteocytes and osteoblasts in response to iron deficiency or hypoxia.^(^ ^35^ ^)^ The degradation of 1,25(OH)2D occurs in the kidneys by CYP24A1, which is stimulated by 1,25(OH)2D3 and FGF23,^(^ ^36^ ^)^ while inhibited by PTH. The mechanisms by which FGF23 is degraded and removed from the circulation are currently unknown, but the kidney is likely degrading and removing FGF23.^(^ ^37^ ^)^

1,25(OH)2D exerts its biological functions by binding to the vitamin D receptor (VDR). After ligand binding, a heterodimer is formed with the retinoid X receptor (RXR), and this VDR‐RXR complex binds to specific genomic sequences (vitamin D response elements [VDRE]) in the promoter region of target genes, resulting in the repression or induction of gene transcription. Apart from its classical effects on calcium and phosphorus homeostasis, vitamin D also exerts non‐classical effects as regulator of (i) proliferation and differentiation, (ii) hormone secretion, and (iii) immune system.^(^ ^38^ ^)^

In humans, the plasma levels of 25OHD, the main circulating form of vitamin D, is used as determinant of vitamin D status due to its long half‐life (15 days).^(^ ^26^ ^)^ Although there is no clear consensus for optimal serum level of 25OHD, it is assumed that 25(OH)D serum levels of 30 ng/mL (75 nmol/L) or even higher are needed for optimal health.^(^ ^39^ ^)^

FGF23 exerts its actions on target tissue through binding to FGF receptors (FGFRs) existing in four different isoforms (FGFR1–4). Enhanced binding affinity of FGF23 to FGFRs is achieved via its specific coreceptor, α‐klotho, which is a transmembrane protein that exists in membrane‐bound form acting as a coreceptor for IGF23^(^ ^40^ ^)^ and as a secreted soluble form found in blood that exerts endocrine and paracrine actions in distant organs.^(^ ^13^, ^41^ ^)^ Although FGFRs are widely expressed in different tissues, α‐klotho expression is restricted to renal tubules and parathyroid gland.^(^ ^14^, ^42^, ^43^ ^)^ But FGF23 can also target cells that lack α‐klotho, and several other cofactors have been proposed as participants in the formation of FGF/FGFR complex.^(^ ^44^, ^45^, ^46^, ^47^, ^48^, ^49^ ^)^ Upon FGF binding and ligand‐induced autophosphorylation of FGFR, distinct downstream signaling pathways are activated according to the type of FGFR isoform and the presence or absence of α‐klotho, leading to cell type–specific events and tissue‐specific effects.^(^ ^10^ ^)^ The primary physiological action of FGF23 is the maintenance of serum phosphate levels within normal range. FGF23 directly targets the kidney via stimulation of the classic FGFR/klotho complexes, to increase phosphate excretion by suppressing the sodium‐dependent phosphate transporter expression in the proximal tubule^(^ ^10^ ^)^ or by inhibiting the secretion of parathyroid hormone,^(^ ^42^ ^)^ which further contributes to an increase in phosphate excretion. Normal values for FGF23 serum levels averaged 34 ± 8 ng/L (39 ± 24 RU/mL) in healthy individuals.^(^ ^50^ ^)^

## 3 Which Cells in the Lung Express VDR, FGFR Isoforms, and/or α‐Klotho for Vitamin D or FGF23 Targeting?

During fetal development, animal studies have revealed the presence of VDR in alveolar type 2 cells during the last quarter of gestation^(^ ^1^ ^)^ and expression of the vitamin D (in)activating enzymes (CYP27B1 and CYP24A1) just before birth.^(^ ^51^ ^)^ After birth, many cells in the lung are expressing VDR and/or vitamin D regulatory enzymes. Actually, in humans, airway epithelial cells express VDR but also high levels of activating CYP27B1 and low levels of CYP24A1 at baseline and they constitutively generate 1,25 dihydroxyvitamin D.^(^ ^2^, ^8^ ^)^ Intriguingly, we recently found that VDR localization was restricted to the apical bronchial epithelial cells while being completely absent in the basal cells of the epithelium and in the vascular endothelial cells.^(^ ^3^ ^)^ Lung fibroblasts also express VDR constitutively^(^ ^4^ ^)^ as do airway smooth muscle cells,^(^ ^5^, ^6^ ^)^ which also express CYP24A1 upon stimulation with 1,25 dihydroxyvitamin D.^(^ ^5^ ^)^ Among the cells engaged in the innate immune response, alveolar macrophages from normal individuals are able to convert 25(OH)D to 1,25 dihydroxyvitamin D only when activated with TLR2/1 ligands, INF‐γ, or LPS,^(^ ^52^, ^53^, ^54^ ^)^ and they express CYP27B1^(^ ^9^ ^)^ and also a nonfunctional CYP24A1 that prevents the catabolism of 25(OH)D and 1,25 dihydroxyvitamin D.^(^ ^55^ ^)^ Mouse data also indicate that VDR is highly expressed in alveolar macrophages, and in response to 1,25 dihydroxyvitamin D3, GM‐CSF‐induced alveolar macrophage proliferation is inhibited.^(^ ^7^ ^)^ There is also evidence that neutrophils express functional VDR to the same extent as monocytes do,^(^ ^56^ ^)^ whereas NK cells express hydroxylases in addition to VDR.^(^ ^57^ ^)^ VDR expression has also been reported in cells releasing inflammatory mediators such as eosinophils^(^ ^58^ ^)^ and mast cells, the latter also expressing CYP27B1 and being able to produce locally 1,25 dihydroxyvitamin D.^(^ ^59^ ^)^ Dendritic cells that form a contiguous network throughout the airway epithelium constitutively express CYP27B1 with a low production of 1,25 dihydroxyvitamin D,^60^, ^61^ ^)^ and VDR expression is downregulated as monocytes differentiate into immature dendritic cells.^(^ ^61^ ^)^ Finally, cells implicated in adaptive immune response such as activated T lymphocytes and B lymphocytes express VDR, and B‐lymphocytes, which also express CYP27B1, are thus able to produce 1,25 dihydroxyvitamin D from 25(OH)D^(^ ^62^, ^63^, ^64^ ^)^ (Table 1). {TBL 1}

**Table 1:** Overview of the Cell Types Expressing Vitamin D Receptor (VDR), 25‐Hydroxylase (CYP24A1), 1α‐Hydroxylase (CYP27B1), or 1,25 Dihydroxyvitamin D (1,25(OH)2D)

Cell types	VDR	CYP24A1	CYP27B1	1,25(OH)2D
Alveolar type 2 cells	X^(^ ^1^ ^)^	X^(^ ^51^ ^)^	X^(^ ^51^ ^)^	
Airway epithelial cells	X^(^ ^2^, ^3^ ^)^ Apically^(^ ^3^ ^)^	X^(^ ^2^, ^3^, ^8^ ^)^	X^(^ ^2^, ^3^, ^8^ ^)^	Constitutively^(^ ^8^ ^)^
Lung fibroblasts	X^(^ ^4^ ^)^			
Airway smooth muscle cells	X^(^ ^5^, ^6^ ^)^	X when stimulated with 1,25(OH)2D^(^ ^5^ ^)^		
Alveolar macrophages	X^(^ ^7^ ^)^	Non‐functional form^(^ ^55^ ^)^	X^(^ ^9^ ^)^	X when stimulated^(^ ^52^, ^53^, ^54^ ^)^
Neutrophils	X^(^ ^56^ ^)^			
Monocytes	X^(^ ^56^ ^)^			
NK cells	X^(^ ^57^ ^)^	X^(^ ^57^ ^)^	X^(^ ^57^ ^)^	
Eosinophils	X^(^ ^58^ ^)^			
Mast cells	X^(^ ^58^ ^)^		X^(^ ^59^ ^)^	X^(^ ^59^ ^)^
Dendritic cells			X^(^ ^60^, ^61^ ^)^	Low^(^ ^60^, ^61^ ^)^
T lymphocytes	X^(^ ^62^ ^)^			
B lymphocytes	X^(^ ^63^ ^)^		X^(^ ^64^ ^)^	X^(^ ^64^ ^)^

Concerning FGF23, human and animal immunostaining studies consistently located its presence in the bronchial epithelial layer,^(^ ^19^, ^65^ ^)^ although the cell types that are precisely expressing FGF23 have not been determined yet. FGF‐23 has also been shown to be expressed in lung tissue of adult mice.^(^ ^12^, ^66^ ^)^ The four FGFR isoforms are expressed in the postnatal but also in the adult murine lung,^(^ ^11^, ^12^ ^)^ and recent human data showed FGFR4 staining localized in the bronchial epithelium,^(^ ^19^ ^)^ while mRNA expression of FGFR1 and FGFR4 was found in bronchial epithelial cells from healthy subjects and particularly from patients with cystic fibrosis.^(^ ^67^ ^)^ Whether the lung expresses α‐klotho is still debated. In humans, it has been shown to be mainly distributed along the airway epithelium.^(^ ^16^ ^)^ However, α‐klotho RNA‐based studies failed to detect klotho expression in mouse, rat, or human lung,^(^ ^12^, ^13^, ^14^, ^15^, ^68^, ^69^ ^)^ while at the protein levels, several studies identified klotho expression in human or mouse lungs and in large airways^(^ ^16^, ^17^, ^18^, ^19^, ^20^ ^)^ as well as in airway epithelial cells^(^ ^16^ ^)^ and alveolar macrophages,^(^ ^21^ ^)^ while others did not.^(^ ^22^, ^23^ ^)^ Validation of the commonly used anti‐klotho antibodies has been questioned,^(^ ^43^ ^)^ and discrepancies might be related to the non‐specificity of the α‐klotho antibodies used in previous studies.^(^ ^24^ ^)^ A recent study using more specific antibodies revealed that the lung does not express endogenously native α‐klotho protein but derives full‐length α‐klotho from circulation.^(^ ^24^ ^)^ Although it is not well documented which cell types express FGFR isoforms, α‐klotho or FGF‐23 in the lungs, primary bronchial epithelial cells isolated from healthy subjects, COPD or cystic fibrosis patients, and also primary lung fibroblasts obtained from healthy individuals and IPF patients have been shown to respond to exogenous FGF23 and/or α‐klotho.^(^ ^19^, ^65^, ^67^ ^)^

## 4 Is Fetal Lung Development a Potential Target for Vitamin D or FGF23?

The VDR presence in the lung during the last quarter of gestation^(^ ^1^, ^70^ ^)^ and the upregulation of the vitamin D regulatory enzymes just before birth^(^ ^51^ ^)^ support a potential role of vitamin D in the late stage of normal fetal lung development and more particularly a specific role for 1,25 (OH)2D3 in lung maturation. Indeed, VDR appearance during gestation coincided with the time of type 2 pneumocyte differentiation start and surfactant secretion onset, typical events of lung maturation.^(^ ^70^ ^)^ Moreover, in vitro studies on cultures of rat fetal lung explants and freshly isolated cells indicated that exogenous 1,25 (OH)2D3 accelerated the functional maturation of the type 2 pneumocytes through reduction in their glycogen content and increase in their surfactant synthesis and secretion.^(^ ^71^, ^72^ ^)^ Association between VDR expression and individual stages of type 2 cell maturation further confirmed a physiological role of 1,25‐(OH)2D3 during type 2 pneumocyte maturation while showing that 1,25‐(OH)2D3 was active at all stages of differentiation but especially at the intermediate stage corresponding to the onset of decrease in glycogen content.^(^ ^1^ ^)^ The role of 1,25 (OH)2D3 in perinatal lung maturation was further underlined by in vitro and in vivo animal studies showing that 1,25 (OH)2D3 stimulated key alveolar epithelial‐mesenchymal interactions and modulated interstitial lung lipofibroblast proliferation/apoptosis responsible for alveolar thinning.^(^ ^73^ ^)^ Alveolar type 2 cells were actually identified as specific targets for 1,25‐(OH)2D3 during fetal lung maturation as these cells expressed functional VDR and responded to exogenous 1,25 (OH)2D3 by upregulating VDR expression.^(^ ^74^ ^)^ Interestingly, while fetal lung fibroblasts did not express VDR, they were able to convert 25(OH)D3 into 1,25(OH)2D3 in contrast to alveolar type II cells.^(^ ^74^ ^)^

Importantly, the fetus has no endogenous production of 25(OH)D and, actually, only 25(OH)D passes the placenta while 1,25(OH)2D is thought to be de novo produced in the placenta and fetus.^(^ ^75^ ^)^ During pregnancy, the fetus is therefore completely dependent on the mother's vitamin D level with maternal 25(OH)D level representing an important determinant of the fetal 25(OH)D level, also influencing the fetal 1,25(OH)2D level.^(^ ^76^, ^77^ ^)^ The transfer of 25(OH)D via the placenta essentially occurs during the last trimester, meaning that preterm infants are particularly at risk of vitamin D deficiency.^(^ ^78^ ^)^ The impact of low maternal vitamin D levels during pregnancy on fetal lung development and maturation and postnatal consequences for susceptibility to lung diseases is currently being investigated. This issue is highly relevant knowing that hypovitaminosis D is frequent in pregnant women and in neonates^(^ ^79^, ^80^, ^81^ ^)^ (Table 2). {TBL 2}

**Table 2:** Overview of the Actions of Vitamin D, FGF23, and α Klotho in the Lung

Lung	Vitamin D	FGF23	α klotho
Fetal development	Lung maturation^(^ ^1^, ^51^, ^70^, ^71^, ^72^, ^73^, ^74^ ^)^	Structural integrity^(^ ^82^, ^83^ ^)^	Structural integrity^(^ ^84^ ^)^ Anti‐senescence^(^ ^84^ ^)^
Innate/adaptive immunity	Positive modulation^(^ ^85^, ^86^, ^87^ ^)^	Not known	Not known
Inflammation	Anti‐inflammatory^(^ ^5^, ^56^, ^88^, ^89^, ^90^, ^91^ ^)^	Pro‐inflammatory^(^ ^19^, ^67^ ^)^	Anti‐inflammatory^(^ ^16^, ^19^, ^67^ ^)^
Oxidative stress	Antioxidant^(^ ^91^, ^92^, ^93^, ^94^ ^)^	No action	Antioxidant^(^ ^16^, ^23^ ^)^
Infection	Modulation phagocytosis^(^ ^95^, ^96^, ^97^, ^98^, ^99^, ^100^ ^)^ Antibacterial^(^ ^101^, ^102^ ^)^ Antiviral^(^ ^2^, ^8^, ^88^, ^103^, ^104^, ^105^, ^106^ ^)^ Antimicrobial^(^ ^90^, ^101^, ^102^, ^107^, ^108^ ^)^	Not known	Not known
Remodeling/damage	Antifibrotic^(^ ^4^, ^109^, ^110^, ^111^, ^112^ ^)^ Antiproliferative^(^ ^113^, ^114^ ^)^ Antiprotease^(^ ^113^, ^114^ ^)^	Antifibrotic^(^ ^65^ ^)^	Antifibrotic^(^ ^65^ ^)^
Epithelial barrier	Maintenance integrity^(^ ^115^, ^116^, ^117^, ^118^ ^)^	Not known	Not known

Several birth cohort studies have shown that maternal vitamin D deficiency is associated with postnatal impairment in lung function in childhood,^(^ ^119^, ^120^, ^121^ ^)^ with increased risk for developing asthma,^(^ ^122^, ^123^ ^)^ which is further emphasized by a recent meta‐analysis including 16 studies.^(^ ^124^ ^)^ Moreover, in full‐term infants, low levels of 25‐hydroxyvitamin D in cord blood were shown to be associated with poor lung function performance assessed using infant lung function testing and increased respiratory infection in infancy.^(^ ^125^ ^)^ Rodent models confirmed the causal role of maternal vitamin D deficiency in promoting abnormal airway and alveolar development, altered lung function, and airway hyperreactivity in offspring.^(^ ^126^, ^127^, ^128^, ^129^, ^130^ ^)^ Whole lung transcriptome alterations in progenies from maternal vitamin D–deficient animals indicated transcript level changes related to abnormal lung growth/development and activation of innate immunity and stimulation of inflammation.^(^ ^131^ ^)^ Taken together, these data suggest that infants born from vitamin D–deficient mothers or preterm infants are at risk for postnatal reduction in lung function with exaggerated inflammatory response to postnatal stimuli. These findings underline the importance of vitamin D action antenatal in the lung and emphasize the postnatal long‐term consequences of failure in vitamin D to exert its action.

By contrast, it is not yet known whether fetal lung can be a target of FGF23 as data in the literature are missing. However, gene deletion of either *fgf23* or *klotho* highlighted the relevance of these genes for proper lung development and also functioning after birth. Indeed, fgf23 null mice develop emphysema,^(^ ^82^, ^83^ ^)^ which appears as early as 3 weeks of age^(^ ^82^ ^)^ and resembles emphysema reported in aged population consistent with premature aging like phenotypes of these mice. This process was shown to be partly mediated by vitamin D. Further, lung histology of homozygous mutant klotho (KL^−/−^) mice resembled that of wild‐type littermates up to 2 weeks of age.^(^ ^84^ ^)^ However, the first emphysematous signs started appearing at 4 weeks of age and progressed with age until premature death around the age of 8 to 10 weeks.^(^ ^84^ ^)^ Structural changes were associated with altered lung function characterized by a longer expiratory time and a higher dynamic compliance at tidal breathing, while arterial oxygen and carbon dioxide partial pressure levels were normal.^(^ ^84^ ^)^ These data indicate that the emphysematous alterations in KL^−/−^ mice are not due to a developmental defect or hypoplasia of the lung but resulted from a progressive destruction of normal alveolar architecture after normal lung development.^(^ ^84^ ^)^ Taken together, these data show that FGF23 plays an important role in lung premature aging and that klotho gene expression is essential for the maintenance of pulmonary integrity during postnatal life.

## 5 Lung Innate and Adaptive Immune System as a Target for Vitamin D or FGF23?

As detailed later in this review, 1,25‐dihydroxyvitamin D can modulate the innate immune system in different ways, notably by induction of antimicrobial peptides after activation of toll‐like receptors but also by altering redox homeostasis and stimulating autophagy. It can also directly affect cells engaged in the innate immune response while inducing proliferation and differentiation of monocytes into macrophages,^(^ ^85^, ^86^, ^87^ ^)^ but whether it increases^(^ ^132^, ^133^ ^)^ or inhibits^(^ ^134^, ^135^ ^)^ the activity of NK cells is still controversial. Conflicting reports might be related to the fact that different types of NK cells including primary NK cells and cell lines were used in these studies. The only available study in vivo indicated that vitamin D supplementation enhanced splenic NK cell activity in mice likely through alteration of the splenic NK cell population.^(^ ^136^ ^)^ In vitro, 1,25‐dihydroxyvitamin D inhibits toll‐like receptor 2 and 4 expression on monocytes, leading to reduced TNF‐α production,^(^ ^137^, ^138^ ^)^ while in murine model, vitamin D deficiency affects macrophage function as shown by impaired chemotaxis and phagocytosis and enhanced pro‐inflammatory cytokine production^(^ ^139^ ^)^ (Table 2).

By contrast, 1,25‐dihydroxyvitamin D exerts an inhibitory action on the adaptive immune response at several levels. It inhibits dendritic cell maturation and differentiation,^(^ ^61^ ^)^ impaired antigen presentation, while also decreasing MHC class II, CD40, CD80, and CD86 costimulatory molecules,^(^ ^140^, ^141^ ^)^ resulting thereby in a tolerogenic phenotype. It also suppresses IL‐12 and enhances IL‐10 production in dendritic cells, which leads to a decreased T‐cell activation.^(^ ^140^ ^)^ Direct effects of 1,25‐dihydroxyvitamin D on T cells include induction of Treg,^(^ ^142^ ^)^ inhibition of Th1 cell proliferation, differentiation with reduction of their IL‐2, IFN‐γ, and TNF‐α production,^(^ ^143^ ^)^ and stimulation of Th2‐cell formation with increased production of IL‐4, IL‐5, and IL‐10.^(^ ^144^ ^)^ These preferential alterations in Th1 functions by 1,25‐dihydroxyvitamin D further promote T‐cell differentiation toward a Th2 phenotype. B cells are indirectly affected by 1,25‐dihydroxyvitamin D through consequences of T‐cell function impairment but also directly with 1,25‐dihydroxyvitamin D, causing inhibition of proliferation and plasma‐cell differentiation, reduction in immunoglobulin secretion and memory B‐cell generation, and induction of B‐cell apoptosis.^(^ ^64^ ^)^

Importantly, locally generated 1,25‐dihydroxyvitamin D in the lung can act in an autocrine or paracrine fashion to exert its immunomodulatory functions, contributing thereby to the regulation of pulmonary immune responses. Expression of CYP27B1 in immune cells is controlled by immune signals with a close link between TLR ligation^(^ ^145^, ^146^ ^)^ or cytokine secretion^(^ ^145^, ^146^, ^147^, ^148^ ^)^ and the expression of CYP27B1 and VDR, notably in human bronchial epithelial cells^(^ ^8^, ^149^ ^)^ and alveolar macrophages.^(^ ^54^ ^)^ In addition, in contrast to the renal CYP27B1 regulation, CYP27B1 expression in macrophage is not suppressed by 1,25‐dihydroxyvitamin D and there is no negative feedback by 1,25‐dihydroxyvitamin to control its own production.^(^ ^139^ ^)^ As mentioned earlier, macrophages express a catabolically dysfunctional form of CYP24A1 that is unable to degrade 1,25‐dihydroxyvitamin D,^(^ ^55^ ^)^ which suggests that overproduction of 1,25‐dihydroxyvitamin D in macrophages might promote innate effector functions. However, the CYP27B1 in macrophage is highly substrate‐driven,^(^ ^150^ ^)^ meaning that serum 25(OH)D bioavailability to macrophages is a key determinant of normal/abnormal physiological control of innate and adaptive immunity. This is supported by in vitro data indicating that failure of the innate immune function of the macrophage can be rescued by exchanging vitamin D–deficient with vitamin D–sufficient human serum in the microenvironment of the macrophages.^(^ ^151^ ^)^ Whether CYP27B1 enzyme in other immune cells is also depending on serum 25(OH)D bioavailability to locally produce 1,25‐dihydroxyvitamin D has not yet been established, although this is likely to be the case. Indeed, apart from macrophages, airway epithelial cells are also able to increase the machinery to convert locally 25(OH)D to 1,25‐dihydroxyvitamin D in response to pathogens to directly increase VDR‐regulated genes implicated in recognition and killing of pathogens such as TLR coreceptor CD14 and antimicrobial peptide cathelicidin.^(^ ^8^ ^)^ In case of viral infection, locally produced 1,25‐dihydroxyvitamin D by airway epithelium modulates inflammatory chemokine and cytokine expression, reducing thereby the innate antiviral response but without affecting viral replication.^(^ ^88^ ^)^

Finally, no studies so far have investigated whether lung innate and adaptive immune system could be a target for FGF23.

## 6 Lung Inflammation as a Target for Vitamin D or FGF23?

There are consistent evidences indicating that several key inflammatory pathways in the lungs are potential targets for vitamin D action. In particular, 1,25‐dihydroxyvitamin D blocks NF‐κB activity by inhibiting nuclear translocation of its complex in cells such as macrophages and airway epithelial cells while upregulating IĸBα expression^(^ ^88^, ^152^, ^153^ ^)^ or through direct interaction between VDR with IĸB kinase β blocking, thereby, the canonical NF‐ĸB activation pathway.^(^ ^154^ ^)^ In addition, by inhibiting NF‐κB binding to DNA, 1,25‐dihydroxyvitamin D hinders the transcriptional activity of NF‐κB in lung fibroblasts.^(^ ^155^ ^)^ In human lymphocyte, it also downregulates the levels of p50, p105 precursor, and c‐rel, decreasing, thereby, their transcriptional activity.^(^ ^156^ ^)^ 1,25‐dihydroxyvitamin D also inhibits p38 MAPK pathway in monocytes by inducing MAPK phosphatase‐1, which inhibits p38 activation by dephosphorylation^(^ ^157^ ^)^ (Table 2).

The anti‐inflammatory action of vitamin D is further emphasized by in vitro studies in cells derived from animal models and humans showing reduction in pro‐inflammatory cytokine and chemokine production in different cells exposed to various stimuli upon treatment with vitamin D. These cells include dendritic cells,^(^ ^89^ ^)^ neutrophils,^(^ ^56^ ^)^ alveolar macrophages,^(^ ^90^ ^)^ airway epithelial cells,^(^ ^88^, ^91^ ^)^ and airway smooth muscle cells.^(^ ^5^ ^)^ In vivo data on the relevance of vitamin D as an anti‐inflammatory agent in the lungs are mainly issued from animal models of vitamin D deficiency. In an allergic airway disease mouse model, vitamin D deficiency was shown to worsen neutrophilic influx in a sex‐specific fashion when a low‐dose model of allergen sensitization was used.^(^ ^158^ ^)^ Similarly, in mice infected with Aspergillus fumigatus, vitamin D deficiency aggravated and prolonged lung inflammation and enhanced broncho‐alveolar lavage cell counts that was dominated by neutrophils after Aspergillus fumigatus inoculation.^(^ ^159^ ^)^ We also found that vitamin D deficiency in mice exacerbated airway and lung inflammation upon cigarette smoke exposure, while increasing airway neutrophilia, the neutrophil chemoattractant KC (mouse homolog of IL‐8) and TNF‐α in the broncho‐alveolar fluid.^(^ ^160^ ^)^ In addition, deletion of VDR in mice is associated with an increased inflammatory cell influx in the airways with elevated levels of MCP‐1 and KC in the lungs.^(^ ^161^ ^)^ Interestingly, intratracheal or peroral administration of 1,25(OH)2D to hamsters was shown to inhibit the recruitment of neutrophils after LPS inhalation, the effect being stronger with intratracheal than peroral administration.^(^ ^162^ ^)^ We also found that local administration of 1,25(OH)2D directly to the lungs reduced the neutrophilic infiltration caused by intratracheal instillation of LPS in mice and it also normalized KC and IL‐6 mRNA levels in LPS‐treated vitamin D–deficient mice (unpublished data). Taken together, these data suggest that local administration of 1,25(OH)2D to the lung may provide an efficient and promising strategy to fight against lung inflammation, although additional research is needed to determine the potential long‐term benefit of such approach.

**Fig 2:**
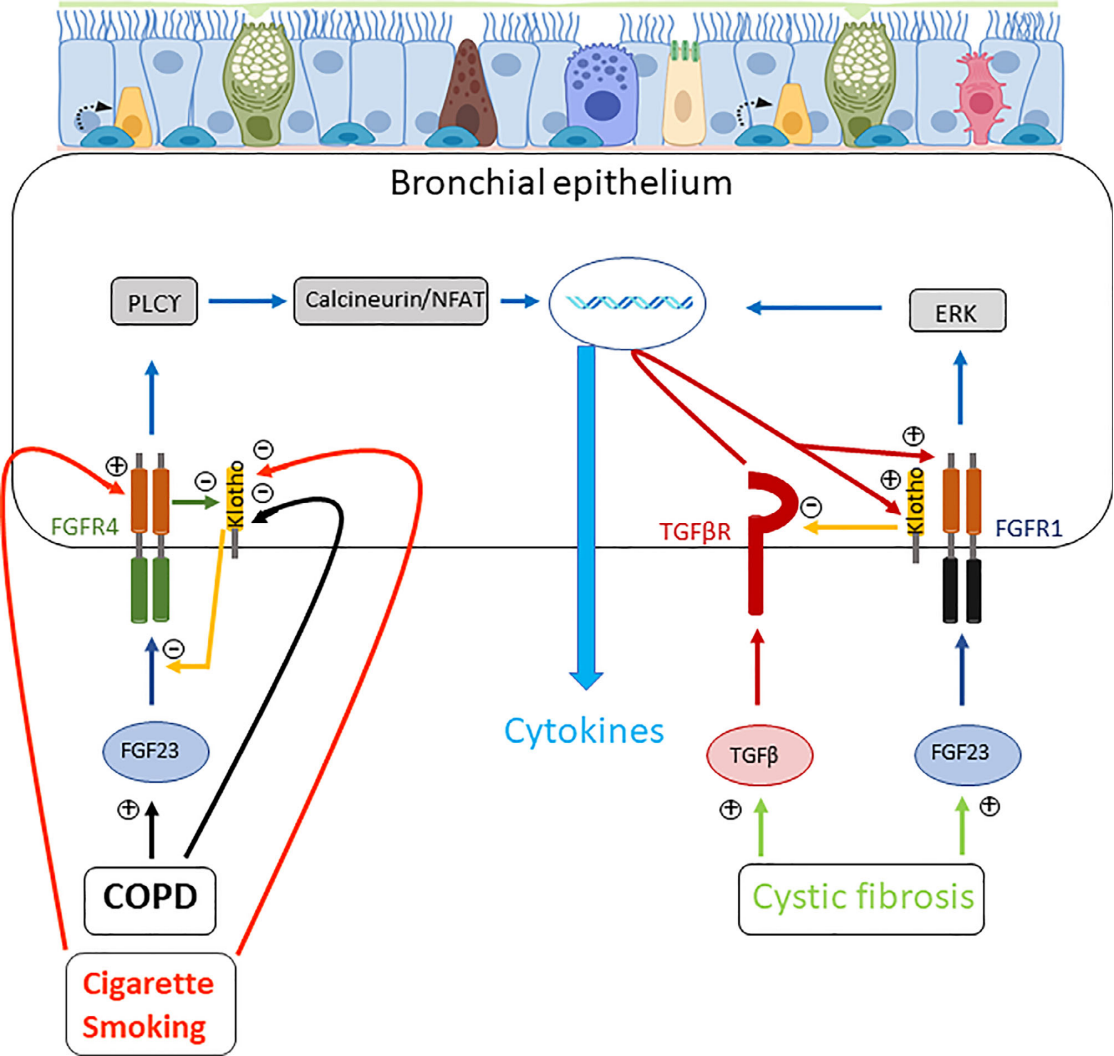
Schematic diagram depicting the effects of FGF23 and α‐klotho on cytokine production in bronchial epithelial cells in context of COPD, cigarette smoking, or cystic fibrosis. Drawn from data described in Krick and colleagues.^(^ ^19^, ^67^ ^)^ Icons have been designed by Biorender. See text for details.

Several studies have highlighted the role of FGF23 in promoting lung inflammation and the anti‐inflammatory properties of α‐klotho. First, it is intriguing that plasma levels of FGF23 are elevated in lung diseases characterized by chronic inflammation such as cystic fibrosis^(^ ^67^ ^)^ and COPD.^(^ ^19^ ^)^ In COPD patients, enhanced plasma FGF23 coincided with elevated inflammatory cytokine in lung and circulation and was positively correlated to IL‐6 serum levels.^(^ ^19^ ^)^ Surprisingly, FGF23 levels were not related to disease severity, suggesting that the reduced FGF23/klotho dysregulation in advanced disease might be due to a kind of “burnout” when residual mass of viable lung epithelium decreases.^(^ ^19^ ^)^ As such, FGF23/klotho perturbations might serve as a disease marker but less as a disease severity marker.^(^ ^19^ ^)^ Furthermore, in combination with cigarette smoke, FGF23 directly targeted COPD human bronchial epithelial cells to induce IL‐1β secretion through activation of FGFR4 and the pro‐inflammatory PLCϒ/calcineurin/NFAT signaling pathway^(^ ^19^ ^)^ (Fig. 2), {FIG2} a pathway known to mediate the pathophysiological actions of FGF23.^(^ ^163^, ^164^ ^)^ Finally, in cystic fibrosis patients, the elevated FGF23 plasma levels synergistically induced TGF‐β mediated airway inflammation (Fig. 2).^(^ ^67^ ^)^ Although these data support a pro‐inflammatory role of FGF23 in lung diseases, there are several evidences showing that α‐klotho exerts anti‐inflammatory actions. First, in bronchial epithelial cells from cystic fibrosis patients, klotho overexpression or supplementation was able to reduce TGF‐β and FGF23‐induced IL‐8 secretion while inhibiting Smad 3 phosphorylation (Fig. 2).^(^ ^67^ ^)^ Likewise, knockdown of klotho in 16HBE cells resulted in enhanced mRNA expression of inflammatory cytokines such as IL‐6, IL‐8, and MCP‐1, indicating that endogenous klotho plays a role in the inflammatory process of human bronchial epithelial cells.^(^ ^16^ ^)^ In addition, when combined with cigarette smoke, depletion of klotho intensified the sensitivity of the human bronchial epithelial cells to cigarette smoke–mediated inflammation, with NF‐κB and ERK1/2 signaling pathways being implicated in the immunoinflammatory‐regulatory functions of klotho.^(^ ^16^ ^)^ These data suggest that klotho exerts protective effects against chronic inflammation and may therefore slow down the progression of lung diseases characterized by chronic inflammation. This is particularly relevant knowing that (i) soluble klotho plasma levels are reduced in patients with idiopathic pulmonary fibrosis in whom FGF23 plasma levels are elevated^(^ ^65^ ^)^; (ii) α‐klotho expression is downregulated in lung of healthy smokers and even more so in COPD patients compared with non‐smokers^(^ ^16^ ^)^ as well as in primary interstitial lung fibroblasts of patients with idiopathic pulmonary fibrosis^(^ ^65^ ^)^; and (iii) secreted α‐klotho is reduced after exposure of 16HBE cells or primary bronchial epithelial cells obtained from COPD patients to cigarette smoke,^(^ ^16^, ^19^ ^)^ an effect associated with a dose‐dependent upregulation of FGFR4 in the bronchial epithelial cells of COPD patients.^(^ ^19^ ^)^ These data highlight that soluble klotho protects bronchial epithelial cells of COPD patients from pro‐inflammatory effects of FGF23 and cigarette smoke.^(^ ^19^ ^)^

In vivo studies confirmed the anti‐inflammatory role of α‐klotho and the pro‐inflammatory action of FGF23 while showing that klotho‐deficient mice, which have elevated serum FGF23 levels and enhanced FGF23 mRNA in lungs, presented with enhanced inflammatory cells, especially neutrophils and macrophages in broncho‐alveolar lavage together with elevated levels of IL‐6 mRNA.^(^ ^19^ ^)^ In addition, FGFR4 was shown to be elevated in the lung and upregulated in differentiated tracheal epithelial cells obtained from these mice lacking klotho in which enhanced basolateral IL‐6 secretion was found.^(^ ^19^ ^)^ These results indicate that in the absence of klotho and in the presence of elevated FGF23, activation of FGFR4/PLCγ signaling together with production of inflammatory cytokines are present in the mouse lung.^(^ ^19^ ^)^ Interestingly, overexpression of klotho in mice reduced cigarette smoke–induced IL‐6 upregulation in lung and showed no differences in FGF23 serum levels and cell counts in broncho‐alveolar lavage compared with wild‐type mice.^(^ ^19^ ^)^ Finally, tracheal epithelial cells from mice lacking FGFR4 revealed protection from FGF23 and cigarette smoke–induced IL‐6 increase, an effect associated with a downregulation of klotho expression.^(^ ^19^ ^)^ Of note, airway inflammation with upregulation of neutrophils and monocytes/macrophages in the BAL fluid and upregulation of inflammatory mediators in airway cells were observed in these mice lacking FGFR4 in whom FGF23 mRNA were increased in the lungs.^(^ ^165^ ^)^ This was associated with widened airway spaces and bronchial obstruction,^(^ ^165^ ^)^ typical features of COPD.

These data suggest that inhibition of FGF23 or FGFR4 and stimulation of soluble klotho might serve as a novel anti‐inflammatory strategy in chronic lung diseases.

## 7 Lung Oxidative Stress as a Target for Vitamin D or FGF23?

There are increasing evidences from some in vitro and in vivo studies showing that vitamin D can impact on oxidative stress in lung. Indeed, the production of reactive oxygen species by human airway epithelial cell (16HBE) in response to stimulation with LPS or hydrogen peroxide was shown to be dose‐dependently inhibited by 25(OH)D3.^(^ ^92^ ^)^ In addition, recent transcriptome analysis on primary human bronchial epithelial cells revealed that 1,25(OH)2D upregulated the *G6PD* gene encoding glucose‐6‐phosphate dehydrogenase representing the rate‐limiting step in NADPH generation, which is essential for antioxidant pathways.^(^ ^91^ ^)^ qPCR confirmed the ability for 1,25(OH)2D to enhance G6PD expression in human bronchial epithelial cells treated with 1,25(OH)2D3.^(^ ^91^ ^)^ Interestingly, a dose‐dependent increase in G6PD expression was also observed in 25(OH)D3‐treated human bronchial epithelial cells.^(^ ^91^ ^)^ The latter treatment was associated with an increased ratio of reduced to oxidized glutathione.^(^ ^91^ ^)^ Furthermore, 25(OH)D3 decreased the formation of 8‐isoprostane after exposure to particulate matter.^(^ ^91^ ^)^ Although the number of healthy donors was limited and their vitamin D status was not known, this study provided mechanistic evidence to support a role for vitamin D as antioxidant in the lung (Table 2).

In a mouse model of asthma, vitamin D was shown to normalize the elevated malondialdehyde and reduced activities of superoxide dismutase and glutathione to control levels, while it also increased the levels of NF‐E2‐related factor 2 (Nrf2), a cellular sensor of oxidative stress associated with transcriptional activation of antioxidant‐response element genes.^(^ ^93^ ^)^ Further, heme oxygenase‐1, an endogenous antioxidant and cytoprotective enzyme that may be upregulated by Nrf2, was also enhanced upon vitamin D treatment in this model.^(^ ^93^ ^)^ However, the protective effect of vitamin D in this model was associated with a non‐lethal hypercalcemia that might be avoided when using vitamin D analogue with less calcemic effect. Vitamin D also protected against particles‐induced lung tissue damage through reduction in oxidative DNA damage and cell apoptosis and in addition it regulated the autophagy‐related signals along with activation of Nrf2 transcription factor.^(^ ^94^ ^)^

All together, these findings support a relevant role for vitamin D as antioxidative agent targeting the lung.

FGF23 itself is not implicated in protection against oxidative stress in contrast to α‐klotho, which can directly protect human lung epithelial cells (A459 and primary alveolar type I cells) against oxidative damage and apoptosis induced by hyperoxia and phosphotoxicity through reduction of lipid and protein oxidation and enhancement of endogenous antioxidant activity.^(^ ^23^ ^)^ Likewise, klotho deletion in human bronchial epithelial cells (16HBE) aggravated sensitivity to oxidative stress and exacerbated cellular injury in response to tobacco smoking or oxidative stress, while promoting apoptosis and decreasing cell viability.^(^ ^16^ ^)^ Moreover, oxidative stress response under klotho knockdown was associated with enhanced activation of critical regulatory pathways of oxidative stress, namely Akt/PKB (protein kinase B), p38 MAPK, and ERK1/2, and reduced ability of the cells to induce an endogenous antioxidant response as shown by decreased total nuclear factor erythroid 2‐related factor 2 (Nrf2) expression and increase in its nuclear translocation.^(^ ^16^ ^)^ However, whether these findings would also be observed in human primary lung epithelial cells needs to be determined. Nevertheless, these data clearly support a protective role of klotho against oxidative stress and apoptosis.

## 8 Lung Infection as a Target for Vitamin D or FGF23?

Several in vitro studies in humans and animals have shown that vitamin D is able to improve phagocytic and oxidative burst capacities of macrophages infected with bacteria^(^ ^95^, ^96^, ^97^, ^98^ ^)^ in a dose‐dependent manner.^(^ ^99^, ^100^ ^)^ However, we failed to find such beneficial effects on alveolar macrophages of smokers and non‐smokers stimulated with E. Coli or cigarette smoke.^(^ ^90^ ^)^ Similarly, in healthy individuals, vitamin D was reported to decrease total phagocytic potential of the macrophages and the ability of the individual macrophages to engulf E. Coli, whereas it had no effect on the phagocytic capacity of the neutrophils when challenged with E. Coli.^(^ ^166^ ^)^ These data suggest that although vitamin D is able to modulate the phagocytic and oxidative burst capacities of the macrophages, this effect seems to be related to the pathogen used, the length of the pathogen challenge, and duration of vitamin treatment. Antibacterial properties of vitamin D was further confirmed in several studies showing the ability for vitamin D treatment to promote killing of Gram‐negative bacteria (nontypeable Haemophilus influenzae, Pseudomonas aeruginosa, and Bordetella bronchiseptica) in airway epithelial cells^(^ ^101^, ^102^ ^)^ (Table 2).

Vitamin D has also been shown to exert antiviral properties. In undifferentiated human primary bronchial epithelial cell culture, vitamin D reduced rhinovirus (RV) replication at high concentration (100 to 1000 nM)^(^ ^2^, ^103^ ^)^ and this despite a downregulation of VDR expression.^(^ ^2^ ^)^ In fully differentiated primary human bronchial epithelial cells, however, vitamin D did not affect rhinovirus replication at lower concentration (10 nM).^(^ ^104^ ^)^ The same finding was reported in undifferentiated bronchial epithelial cells infected with respiratory syncytial virus (RSV) despite the use of higher vitamin D concentration (100 nM).^(^ ^88^ ^)^ Vitamin D also reduced RV‐ and RSV‐induced inflammatory response of undifferentiated airway epithelial cells,^(^ ^2^, ^8^ ^)^ while it enhanced RV‐induced IL‐8 secretion and did not affect IL‐6 levels of primary bronchial cells cultured under air liquid interface condition.^(^ ^104^ ^)^ Suppression of inflammation by vitamin D was also reported in influenza (H9N2 and H1N1)‐infected human lung A549 epithelial cells^(^ ^105^, ^106^ ^)^ in which vitamin D also diminished autophagy and restored increased apoptosis.^(^ ^105^ ^)^ Inhibitory effects of vitamin D on poly(I:C)‐induced inflammatory responses were also observed in primary airway epithelial cells.^(^ ^8^, ^149^ ^)^ Recent data in A549 cells also indicated that vitamin D attenuated RV‐induced expression of intercellular adhesion molecule‐1 (the receptor for major group human rhinoviruses) and platelet‐activating factor receptor (a cell surface receptor implicated in adhesion of virulent S. pneumoniae to respiratory epithelial cells) while enhancing the NF‐κB inhibitor IκBα and CAMP, the precursor for the antimicrobial peptide cathelicidin.^(^ ^167^ ^)^

With the recent COVID‐19 pandemic, several studies have been conducted to determine whether vitamin D could efficiently reduce COVID‐19 risk and be used as adjuvant treatment in COVID‐19. Whether vitamin D status was a risk factor or had an impact of COVID‐19 infection severity was also examined. These data have been explored in different systematic reviews with meta‐analysis.^(^ ^168^, ^169^, ^170^, ^171^ ^)^ It seems that vitamin D supplementation might be associated with improved clinical outcomes (ICU admission and/or mortality), especially in patients with moderate to severe COVID‐19 requiring hospitalization. But appropriate dose, duration, and mode of administration warranted future research.^(^ ^168^ ^)^ However, current evidences for an effect of vitamin D supplementation on all‐cause mortality at hospital discharge, decreased need for invasive mechanical ventilation, or higher risk for adverse events are low, but actually only three randomized controlled trials (RCTs) including 356 participants of whom 183 received vitamin D were included in this systematic review.^(^ ^169^ ^)^ Additional well‐designed and adequately powered RCT trials are needed to determine the potential benefit of vitamin D supplementation as treatment in COVID‐19. This is particularly relevant knowing that 25(OH)D levels were shown to be lower in SARS‐CoV‐2‐positive patients than in negative patients, and even lower in patients with severe disease compared with patients with non‐severe course of the disease, as well as in patients who died from COVID‐19 compared with patients who were discharged.^(^ ^170^ ^)^ This study also showed that the risk of severe disease was higher in patients with vitamin D deficiency, but this was not associated with a higher risk of mortality.^(^ ^170^ ^)^ However, in a larger meta‐analysis including 17 studies (compared with nine studies in Crafa and colleagues^(^ ^170^ ^)^), vitamin D deficiency was well associated with higher mortality and also higher rates of hospital admission and longer hospital stays.^(^ ^171^ ^)^ These data definitely underlined the importance of the vitamin D status in COVID‐19, although further research is needed to highlight the potential benefit of vitamin D supplementation in patients with COVID 19. But, it is worth noting that a recent updated meta‐analysis including 56 RCTs indicated that vitamin D supplementation overall reduced the risk for acute respiratory infection when administered daily at a dose of 400 to 1000 IU for up to 12 months, and this treatment regimen was also shown to be safe.^(^ ^172^ ^)^ However, further investigations are required to determine the extent to which these findings might be relevant for COVID‐19.

Finally, vitamin D can also enhance antimicrobial defense by stimulating the production of antimicrobial peptides like cathelicidin (LL‐37). These antimicrobial peptides contain vitamin D–responsive element in the promotor region of their genes and are upregulated upon stimulation with vitamin D.^(^ ^107^ ^)^ Several in vitro studies confirmed that vitamin D enhanced cathelicidin expression in human bronchial epithelial cells (NHBE, 16HBE)^(^ ^102^ ^)^ and primary bronchial epithelial cells obtained from donors^(^ ^101^, ^108^ ^)^ and COPD patients,^(^ ^108^ ^)^ as well as in alveolar macrophage of smokers and non‐smokers and THP1 macrophages.^(^ ^90^ ^)^

These data indicate that lung infection due to bacterial or virus infection is likely to be a potential target for vitamin D that may help clearing pathogens and reduce inflammatory response.

There have been no studies so far investigating whether lung infection could be a target of FGF23 and anti‐infectious properties have not been reported for FGF23 or klotho.

## 9 Lung Remodeling/Damage as a Target for Vitamin D or FGF23?

Airway remodeling is characterized by structural changes in the composition, content, and organization of the airway wall, such as subepithelial fibrosis, increased airway smooth muscle mass, goblet cell hyperplasia, mucous gland hyperplasia, and proliferation of blood vessels. Several studies have consistently reported the potential of vitamin D in improving disease‐induced lung remodeling. In particular, the antifibrotic and antiproliferative effects of vitamin D have been well described on various cell types, and signaling pathways and the current data suggest that vitamin D actions may help in preventing and treating damaging structure changes in the airways (Table 2).

Several in vitro studies have, indeed, shown that vitamin D suppresses airway smooth muscle cell proliferation, mucus, and matrix metalloproteinase by cultured human bronchial cells, while downregulating expression of two known airway remodeling modulators, MMP9 and ADAM33.^(^ ^113^, ^114^ ^)^ It also inhibits the proliferation and activation of TGF‐β1‐treated murine fibroblasts and reduces the secretion of fibrotic mediators like fibronectin and collagen by fibroblasts,^(^ ^4^ ^)^ as well as the TGF‐β‐induced elevation of extracellular matrix genes in human^(^ ^109^ ^)^ and mouse^(^ ^110^ ^)^ bronchial fibroblasts and human alveolar epithelial cells.^(^ ^111^ ^)^ In addition, vitamin D treatment attenuates the increased expression of mesenchymal markers (N‐cadherin and vimentin) and decreased expression of epithelial marker E‐cadherin induced by TGF‐β in human alveolar epithelial cells,^(^ ^111^ ^)^ while it modulates human lung fibroblast‐mediated tissue repair function by regulating PGE2 synthesis and degradation.^(^ ^173^ ^)^ Interestingly, vitamin D treatment also restores VDR expression in TGF‐β treated mouse lung fibroblasts^(^ ^110^ ^)^ and human alveolar epithelial cells.^(^ ^111^ ^)^

In vivo studies confirm that vitamin D supplementation alleviates the features of airway remodeling in animal models of asthma and lung fibrosis by reducing thickness of airway smooth muscle, inhibiting the collagen deposition and α smooth muscle actin mass elevation.^(^ ^93^, ^110^, ^112^, ^174^, ^175^ ^)^ This is accompanied by increased expression of E‐cadherin and decreased vimentin and N‐cadherin expression in the airways^(^ ^174^ ^)^ and by a downregulation of the Wnt/β‐catenin signaling pathway^(^ ^112^ ^)^ and the TGF‐β‐Smad signaling.^(^ ^175^ ^)^ Vitamin D also attenuated particles‐induced lung tissue damage and promoted tissue repair by repressing of TGFβ1 signaling pathway and upregulation of MMP9 expression.^(^ ^94^ ^)^

Taken together, these data suggest that vitamin D may represent an efficient therapeutic agent to limit the detrimental structure changes in the airways.

For FGF23, there have been no studies yet examining the impact of FGF23 on lung remodeling. However, both FGF23 and klotho can alter lung structure because mice lacking FGF23 or klotho develop pulmonary emphysema.^(^ ^14^, ^83^, ^84^ ^)^ Moreover, their combined treatment has been shown to reduce dose‐dependently the TGF‐β‐induced collagen production of primary lung fibroblasts, suggesting that they might protect the lung against fibrosis.^(^ ^65^ ^)^ This is further supported by data in mice showing that klotho overexpression protected against bleomycin‐induced lung fibrosis by reducing collagen deposition that was associated with a reduced collagen production in the fibroblasts of these mice.^(^ ^65^ ^)^ Conversely, klotho‐deficient mice in which FGF23 serum levels are elevated are more susceptible to develop lung fibrosis and display collagen deposition as shown by elevated hydroxyproline content in the lung.^(^ ^65^ ^)^ These data clearly indicate that FGF23 and klotho actions can affect lung structure in different ways.

## 10 Lung Epithelial Barrier Integrity as a Target of Vitamin D or FGF23?

Several studies suggest that vitamin D is likely playing an important role in maintaining epithelial barrier integrity in the lungs. In the bronchial epithelial 16HBE cell line, vitamin D is able to counteract the cigarette smoke–induced epithelial barrier disruption by inhibiting transepithelial electrical resistance reduction, permeability increase, and cleavage of E‐cadherin and β catenin.^(^ ^115^ ^)^ These protective effects were related to downregulation of ERK signaling pathway by vitamin D to attenuate calpain‐1 and thus maintain E‐cadherin.^(^ ^115^ ^)^ Interestingly, vitamin D was also shown to increase expression of cystic fibrosis transmembrane conductance regulator in human bronchial epithelial cells, which may help in promoting epithelial barrier function.^(^ ^116^ ^)^ This finding was also observed after topical delivery of vitamin D in vivo via intranasal administration to mice.^(^ ^116^ ^)^ Data in VDR‐deficient mice showing reduced expression in tight and adherens junction molecule expression, particularly in claudin‐2, −4, −10, −12, and −18, indicate that VDR may play an important role in maintaining pulmonary barrier integrity.^(^ ^117^ ^)^ This is further supported by a mouse study reporting that the vitamin D/VDR signaling attenuated acute lung injury by maintaining the expression of occluding and zonula occludens‐1, preserving thereby alveolar barrier integrity.^(^ ^118^ ^)^ In summary, these studies clearly support a role for vitamin D in promoting epithelial barrier integrity and function (Table 2).

There have been no studies investigating whether FGF23/klotho may affect lung epithelial barrier integrity.

## 11 Conclusion

There is compelling evidence that the lung is a target for vitamin D actions and maybe also for FGF23, although for the latter, more research is needed to determine its impact on the lung. It is now clear that many cells in the lung express VDR and respond to vitamin D, but for FGF23, this still remains to be unraveled. The actions of vitamin D on the lung are essentially beneficial and include immunomodulatory, anti‐inflammatory, anti‐infectious, and antioxidant actions, as well as maintenance of airway structure and epithelial barrier integrity. Despite its multiplicity of functions, several clinical intervention trials with systemic vitamin D supplementation have only shown mild clinical benefits on lung health. One aspect that has not been sufficiently explored yet is the ability for several lung cells to respond to exogenous vitamin D and/or to locally produce active vitamin D that can act in an autocrine or paracrine fashion. Future research should exploit this aspect as local administration of active vitamin D directly to the lungs might be an efficient strategy to stimulate vitamin D actions on lung cells, an effect that might be reinforced through local administration of 25(OH) to favor local production of active vitamin D by lung cells.

Extensive in vitro and animal studies show that FGF23 acts as a harmful agent by promoting inflammation of the lung in different chronic lung diseases. Many of these effects are counterbalanced by klotho, which clearly protects the lung against inflammation, oxidative stress, apoptosis, and potentially against fibrosis. At this stage, it is unknown how this closely interactive FGF23/klotho pathway can be selectively targeted, which is obviously key to develop novel therapeutic interventions.

## Disclosures

The authors state that they have no conflicts of interest.

12

### Peer Review

The peer review history for this article is available at https://publons.com/publon/10.1002/jbm4.10569.
